# Awake endovascular coiling of a dissected intracranial aneurysm in a third-trimester twin pregnancy: A case report

**DOI:** 10.1097/MD.0000000000024239

**Published:** 2021-01-08

**Authors:** Fei Xie, Jianqiang Hao, Seidu A. Richard, Yuanli Yang, Wuchun Zou, Hong-Bin Liu, Min Deng, Changwei Zhang

**Affiliations:** a Department of Neurosurgery; b Department of Obstetrics and Gynecology, Ziyang First People's Hospital, Ziyang; c Department of Neurosurgery, West China Hospital, Sichuan University, Chengdu, P. R. China; d Department of Medicine, Princefield University, Ho-Volta Region, Ghana West Africa.

**Keywords:** anesthesia, aneurysm, awake, coiling, endovascular, hemorrhage, pregnancy

## Abstract

**Rationale:**

Subarachnoid hemorrhages (SAHs) from ruptured intracranial aneurysms are very rare during pregnancy. Management of ruptured intracranial aneurysms with SAH in pregnancy is often challenging because of the risks to the fetus and the mother. We present the first successful awake endovascular coiling of a dissected intracranial aneurysm in a third trimester twin pregnancy.

**Patient concerns:**

A 28 years’ old pregnant woman was admitted at the obstetric department of our hospital on account of very severe headaches associated with nausea and vomiting.

**Diagnosis:**

Emergency obstetric ultrasound scan done confirmed 32 weeks’ twin gestation, whereas magnetic resonance imaging established hemorrhage in the suprasellar cistern and the subarachnoid space. Magnetic resonance angiography revealed a dissected aneurysm in the ophthalmic segment of the left internal carotid artery.

**Interventions:**

Awake cerebral angiography as well as embolization of the aneurysm with coils was done via the transarterial route and the twins were delivered via caesarean section at 37 weeks’ gestation.

**Outcomes:**

Two years’ follow-up indicated no complications and children as well as their mother are healthy.

**Lesions:**

Awake endovascular coiling was very beneficial in our case because we avoided general anesthesia and the use of osmotic diuretics which are potentially hazardous during pregnancy.

## 1 Introduction

The incidence of subarachnoid hemorrhages (SAHs) from ruptured intracranial aneurysms are very rare during pregnancy.^[1–3]^ Nevertheless, maternal death rate from aneurysmal rupture is about 5% to 12%.^[1,2]^ The possibilities of aneurysm formation, progression, as well as rupture may be influenced by normal hemodynamic changes during pregnancy as a result of increase vascular stress.^[3–6]^ Also, hormones like estrogen, progesterone, as well as vascular endothelial growth factor have been implicated as causes of intracranial aneurysm during pregnancy.^[3,7]^

Radiological evaluation may post danger to both the mother and the fetus during pregnancy, nevertheless, their use is very inevitable.^[8,9]^ It is advocated that, during pregnancy, radiological evaluation should be done under lead aprons.^[1]^ The treatment of SAH from aneurysmal rupture in pregnancy varies from conservative, surgical clipping, or endovascular coiling.^[3]^ We present the first successful awake endovascular coiling of a dissected intracranial aneurysm in a third trimester twin pregnancy.

## 2 Case presentation

A 28 years’ old pregnant woman was admitted at the obstetric department of our hospital on account of very severe headaches associated with nausea and vomiting. She was apparently well until the above symptomology started over night. She denied any form of vaginal discharges. She was Gravida 3, 1 live delivery via cesarean section and 1 termination of pregnancy (G3P1+1). Her antenatal history in the index presentation was unremarkable. She was not a hypertensive or a diabetic. On admission, obstetric examination dated her pregnancy at 32 weeks with dual fetal heart beats. Vaginal examination was unremarkable and there were no uterine contractions. General physical examination was unremarkable. Routine laboratory investigations were grossly normal.

**Figure 1:**
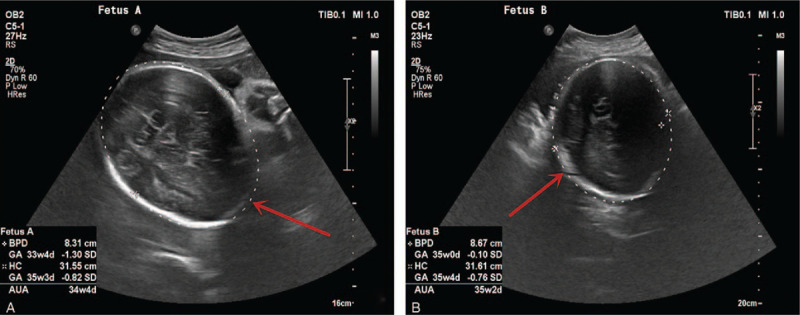
(A and B) Ultrasound scan images showing the fetuses. (A) is the first fetus, whereas (B) is the second fetus. Red arrows.

Emergency obstetric ultrasound scan confirmed 32 weeks’ twin gestation (Fig. 1A and B). Fetal heart beats were present and normal. All membranes were intact. The obstetric team, however, did not initially suspect a central nervous disease. Considering the severity of the symptomology above, the obstetric team initial suspected severe preeclampsia. However, the blood pressure of the patient was normal. This prompted the obstetric team to request for a magnetic resonance imaging (MRI). Also, the patient was started on intramuscular dexamethasone 6 mg 12 hours apart to mature the fetal lungs in preparation for emergency delivery.

**Figure 2:**
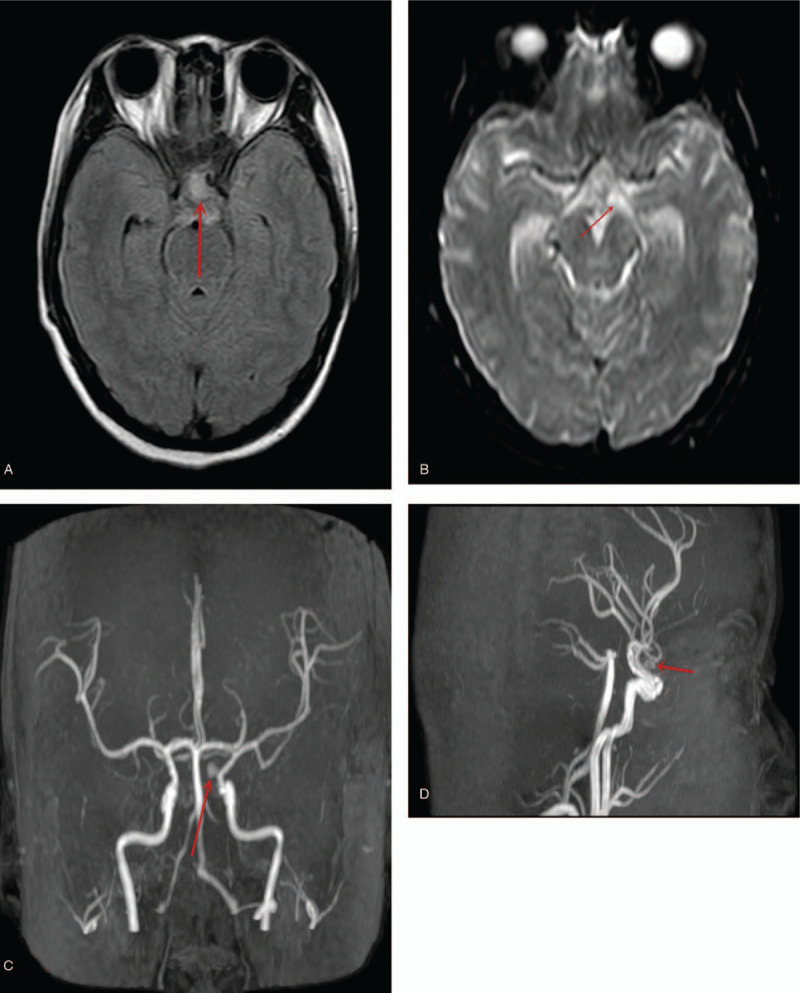
(A) Preoperative magnetic resonance imaging (MRI) showing high signal intensity in suprasellar cistern on FLAIR sequence. (B) Preoperative MRI showing high signal intensity was seen in the subarachnoid space on diffusion-weighted imaging sequence. (C and D) Magnetic resonance angiography images showing a dissected aneurysm in the ophthalmic segment of the left internal carotid artery.

Interestingly, MRI showed high signal intensity in suprasellar cistern on FLAIR sequence (Fig. 2A), whereas on diffusion-weighted imaging sequence, high signal intensity was seen in the subarachnoid space (Fig. 2B) which signifies hemorrhage in the suprasellar cistern and the subarachnoid space. There was no acute hydrocephalus. A few small ischemic lesions in the bilateral frontal and parietal cortexes were also visible. Magnetic resonance angiography (MRA) revealed a dissected aneurysm in the ophthalmic segment of the left internal carotid artery (ICA) (Fig. 2 C and D). Adequate protection of the fetuses from radiation was achieved with the use of regular lead aprons during the MRI and MRA evaluation. After establishing SAH, the patient's neurological state was evaluated again and observed to be H&H grade 2.

**Figure 3:**
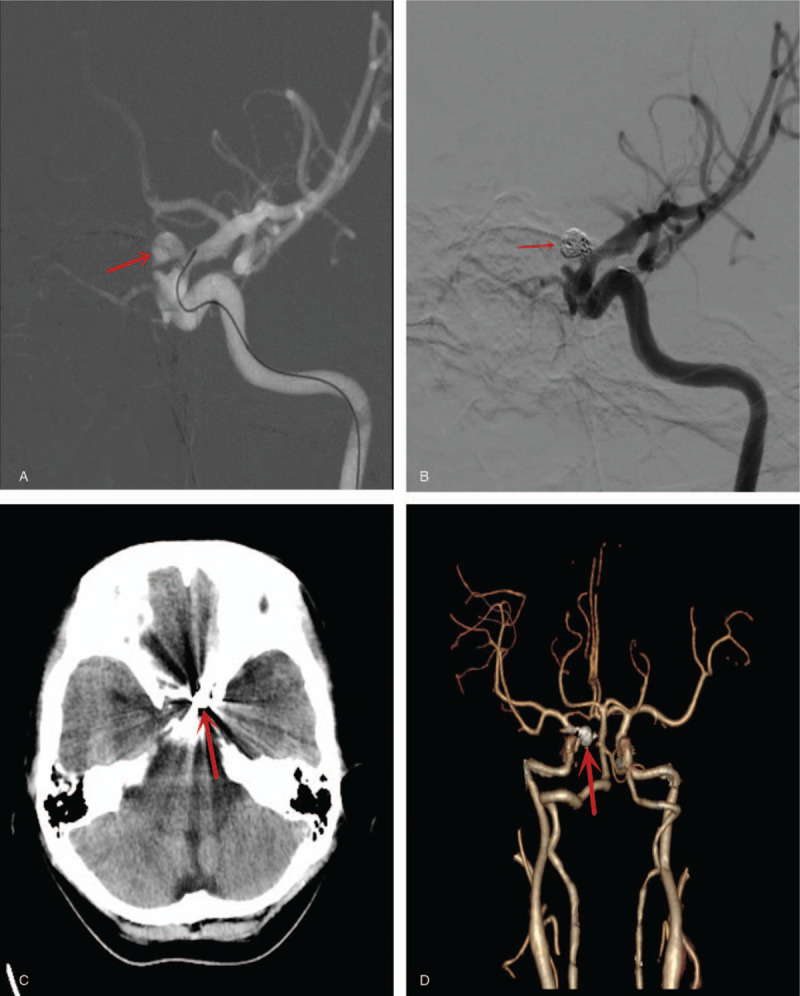
(A) Intraoperative digital subtracting angiography (DSA) image showing a dissected aneurysm in the ophthalmic segment of the left internal carotid artery (ICA) with an irregular neck as well as distal stenosis in the parent artery. (B) Intraoperative DSA showing occlusion of aneurysm with coils. (C) Postoperative computed tomography (CT) showing the opacity of the coils in-situ. (D) Postoperative CT angiography showing total occlusion of the aneurysm will coils.

We opted for an awake endovascular coiling of the aneurysm to keep the mother and fetuses safe. Also, we wanted the lungs of the fetuses to mature before delivery. The entire procedure was an awake one after infiltrating the canular site with local anesthesia before inserting the catheters. No sedatives and analgesics were used. Cerebral angiography as well as embolization of the aneurysm with coils was done via the trans-arterial route. Intraoperatively, digital subtracting angiography (DSA) confirmed a dissected aneurysm (5.72 mm × 4.82 mm × 4.26 mm) in the ophthalmic segment of the left ICA with an irregular neck as well as distal stenosis in the parent artery (Fig. 3A). The aneurysm was successfully embolized with detectable coils (Stryker; Neurovascular, Fremont, CA) (Fig. 3B). The entire procedure lasted for only 20 minutes without general anesthesia and the use of osmotic diuretics. Postoperative computer tomography (CT) and CT angiogram revealed total occlusion of the aneurysm (Figure 3C and D).

**Figure 4:**
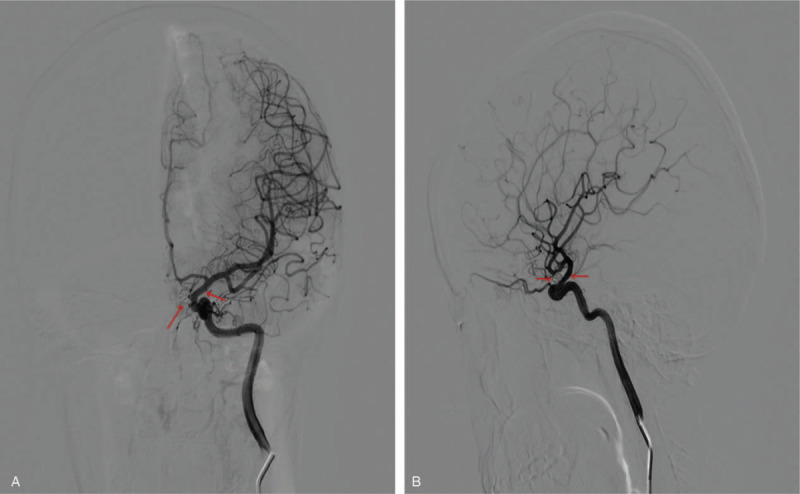
(A and B) Follow-up digital subtracting angiography showing complete embolization of the aneurysm and massive improvement in the distal stenosis.

Adequate protection of the fetuses from radiation was achieved with the use of regular lead aprons during the endovascular procedure. Also, the use of contrast agent (140 mg/mL iohexol injection) was minimal throughout the entire procedure to minimize contrast toxicity to mother and the fetuses. She had no neurological deficits after the operation. She was nurse in ward for 5 weeks before delivery of the fetuses. The fetuses were successfully delivered via cesarean section when the gestation of fetuses reached 37 weeks. The babies and their mother are healthy with no complications. They were discharged home a week after delivery. Also, DSA on first scheduled visit after discharge revealed complete embolization of the of the aneurysm and massive improvement in the distal stenosis (Fig. 4A and B). Two years’ follow-up indicated no complications and the children as well as their mother are healthy.

## 3 Discussion

Intracranial aneurysms in pregnancy are very rare and have been reported in all child bearing age groups but predominantly in pregnant women who are about 30 years and older.^[2,4–6,10,11]^ Pregnancy-related aneurysmal rupture occurs in about 50% of women under the age of 40 years.^[12,13]^ Although aneurysmal rupture in pregnancy has been reported in all the trimesters, most of them were detected in the third trimester.^[14]^ Hunts et al^[14]^ observed about 6% of aneurysmal rupture in the first trimester, whereas about 31% and 55% ruptured in the second and third trimesters respectively and about 8% after delivery. Nevertheless, Barbarite et al^[3]^ in a systematic review established 8%, 11%, and 78% of aneurysms ruptured during the first, second, and third trimesters respectively. We report the first case of an aneurysmal rupture at third trimester twin pregnancy in a 28 years’ old woman.

Changes in physiologic dynamics like hormones, hemodynamic, coagulation, as well as vessel wall have been implicated as pathophysiology of new aneurysm formation and/or weakening of preexisting aneurysms in pregnancy.^[2,12,13,15]^ Hormones like estrogen, progesterone, as well as prostacyclin affect the vascular smooth muscle resulting in a decrease in vascular resistance.^[1,15]^ Also, high levels of relaxin and increased wall tension from intraparenchymal artery hypoplasia may be responsible for aneurysm progression and subsequently rupture during pregnancy.^[3,16]^ Cardiac output increases during the third trimester of pregnancy as a result of increased blood volume to a maximum level of about 1700 mL as compared to nonpregnant women.^[1,15]^ Increment in cardiac output may be responsible for aneurysm progression and subsequently rupture during pregnancy.^[1,15]^ In multiple fetuses as compared to a single fetus, the changes in physiologic dynamics increase even more resulting in the rupture of the aneurysm.

SAH from a ruptured of aneurysm is often associated with high rate of mortality and morbidity.^[1,5]^ Case fatality from SAH during pregnancy is as high as 83%, but most patients recover with prompt diagnosis and treatment.^[3,10,17]^ Also, SAH from aneurysmal rupture is the third most common nonobstetric cause of death during pregnancy.^[1,2,5,14,18]^ A severe and sudden onset of headache is the hallmark of aneurysmal SAH. Patient often describe this type of headache as “an explosion within the head,” “as if something ruptured inside my head,” “the worst headache of my life,” or “as if someone hit my head with a sharp object,”^[5,19]^ The headache, which is typically suboccipital or frontal in nature, is often associated with nausea, vomiting, blurring of vision, neck stiffness, as well as photophobia.^[5,20]^ The cardinal symptomatology of our patient was headaches, nausea, and vomiting. Most patients are often misdiagnosed as severe preeclampsia or eclampsia.^[1,2,5,14,18]^ Nevertheless, the primary differential diagnosis of aneurysmal SAH includes, eclampsia, meningitis, encephalitis, pituitary apoplexy, dural sinus thrombosis, ischemic stroke, intracranial tumors, as well as demyelinating diseases.^[8,9,11]^

CT and MRI are the initial radiological modalities in evaluating cerebral lesions in pregnant women.^[5,9,11,19]^ Nevertheless, cerebral angiogram is the criterion standard radiological modality for detecting intracranial aneurysms.^[1,9]^ The exposure of fetus to radiation is usually the main concern during radiological imaging in pregnancy.^[1]^ MRI was capable of detecting hemorrhage in the suprasellar cistern and the subarachnoid space in our patient. Also, MRA revealed a dissected aneurysm in the ophthalmic segment of the left ICA. Nevertheless, intraoperative angiography confirmed the diagnosis of a dissected aneurysm.

However, the maximum radiation exposure allowed during pregnancy varies in the first, second, and third trimesters.^[1]^ A maximum of up to 0.5 rem radiation exposure is often advocated in the third trimester.^[1]^ It is advocate that, to minimize the exposure of the fetus to radiation, lead aprons must be fitted over the uterus. Furthermore, the exposure to radiation during CT evaluation should be <0.05 rem, whereas exposure during angiography should be <0.1 rem.^[1]^ We utilized the above radiation parameters during the evaluation and treatment of our patient. It is well established that contrast agents have no effect on fetus; nevertheless, excessive usage of contrast agent may result in transient hypothyroism. Also, dehydration of the fetus is often associated with the use of contrast agents.^[1,2]^ Therefore, the usage of contrast agent was very minimal in our case.

Treatment of ruptured intracranial aneurysms with SAH in pregnancy is often challenging because of the risks to the fetus and mother.^[3,17,21]^ Conservative treatment, surgical clipping, or endovascular coiling is main treatment option for ruptured intracranial aneurysms with SAH in pregnancy.^[3,21]^ Medical or conservative treatment with mannitol, an osmotic diuretic, is often avoided because it induces hypovolemia and hypotension in maternal body, which may result in uterine hypoperfusion, and thus result in fetal hypernatremia as well as hyperosmolarity.^[1,2]^ Barbarite et al^[3]^ in systematic review revealed that coil embolization was used more often than surgical clipping (56% vs 36%).

Surgery clipping is very invasive and may require the use of anesthesia and osmotic diuretics after the operation.^[3,8]^ Considering the fact that our patient was carrying twins, we did not opt for surgical clipping because of the associated risks. Studies have shown that, endovascular coiling is advantageous because of shorter operating times as well as shorter hospital stays compared to surgical clipping.^[3,22]^ Therefore, since our patient was carrying twins, it was more advantageous to perform endovascular coiling. The entire procedure lasted for only 20 minutes without general anesthesia and the use of osmotic diuretics. Thus, this is the first successful case of awake endovascular coiling of a ruptured aneurysm in twin pregnancy.

It is advocated that during endovascular procedure in pregnancy, fetus must have minimal exposure to anesthesia because of permanent neurological deficits to the baby.^[3,8,17]^ Also, the use of anticoagulation medications is potentially dangerous to the fetus and also increases bleeding tendencies if cesarean section is required in the shortest possible time.^[3,4]^ After successful occlusion of the aneurysm, we decided to wait up to a month before performing the cesarean section by which time the bleeding tendencies associated with the use of anticoagulation medication during the endovascular produce would have resolved. Several authors have suggested that surgical clipping or endovascular coiling ought to be performed immediately before or after delivery if the aneurysms were detected near term.^[3,17,23]^ It is advocated that vaginal delivery is avoided in pregnant women with unruptured and ruptured intracranial aneurysms with SAH because of the associated mode of anesthesia delivery.^[3,21]^ Also, high-pressure valsalva maneuvers are often needed by the patient which could worsen existing aneurysm-associated complications such as cranial defects from prior clipping.^[3,21]^ Thus, to be at the safest side, we delivered the fetuses via cesarean section when the gestation of the pregnancy reached 37 weeks.

## 4 Conclusions

Awake endovascular coiling was very useful in our case because we avoided general anesthesia and the use of osmotic diuretics which are potentially hazardous during pregnancy. Also, the entire procedure lasted for only 20 minutes with minimal usage of contrast agents which could result in dehydration in the fetuses. In all radiological evaluation during pregnancy, we advocate that the gravid uterus should be adequately protected with regular lead aprons to minimize exposure of the fetus to radiations.

## Author contributions

**Conceptualization:** Fei Xie, Jianqiang Hao, Seidu A Richard, Yuanli Yang, Wuchun Zou, Hong-Bin Liu, Min Deng, Zhang Changwei.

**Data curation:** Fei Xie, Jianqiang Hao, Seidu A Richard, Yuanli Yang, Wuchun Zou, Hong-Bin Liu, Min Deng, Zhang Changwei.

**Formal analysis:** Fei Xie, Jianqiang Hao, Seidu A Richard, Yuanli Yang, Wuchun Zou, Hong-Bin Liu, Min Deng, Zhang Changwei.

**Investigation:** Fei Xie, Jianqiang Hao, Wuchun Zou.

**Methodology:** Fei Xie, Jianqiang Hao, Seidu A Richard, Yuanli Yang, Wuchun Zou, Hong-Bin Liu, Min Deng, Zhang Changwei.

**Resources:** Fei Xie, Yuanli Yang, Hong-Bin Liu, Min Deng, Zhang Changwei.

**Supervision:** Zhang Changwei.

**Writing – original draft:** Seidu A Richard.

**Writing – review & editing:** Fei Xie, Jianqiang Hao, Seidu A Richard, Yuanli Yang, Wuchun Zou, Hong-Bin Liu, Min Deng, Zhang Changwei.
